# Translational Systems Biology of Inflammation

**DOI:** 10.1371/journal.pcbi.1000014

**Published:** 2008-04-25

**Authors:** Yoram Vodovotz, Marie Csete, John Bartels, Steven Chang, Gary An

**Affiliations:** 1 Department of Surgery, University of Pittsburgh, Pittsburgh, Pennsylvania, United States of America; 2 Center for Inflammation and Regenerative Modeling, McGowan Institute of Regenerative Medicine, University of Pittsburgh, Pittsburgh, Pennsylvania, United States of America; 3 Department of Anesthesiology, Emory University School of Medicine, Atlanta, Georgia, United States of America; 4 Immunetrics Corporation, Pittsburgh, Pennsylvania, United States of America; 5 Department of Surgery, Northwestern University, Chicago, Illinois, United States of America; National Center for Biotechnology Information (NCBI), United States of America

## Abstract

Inflammation is a complex, multi-scale biologic response to stress that is also required for repair and regeneration after injury. Despite the repository of detailed data about the cellular and molecular processes involved in inflammation, including some understanding of its pathophysiology, little progress has been made in treating the severe inflammatory syndrome of sepsis. To address the gap between basic science knowledge and therapy for sepsis, a community of biologists and physicians is using systems biology approaches in hopes of yielding basic insights into the biology of inflammation. “Systems biology” is a discipline that combines experimental discovery with mathematical modeling to aid in the understanding of the dynamic global organization and function of a biologic system (cell to organ to organism). We propose the term *translational systems biology* for the application of similar tools and engineering principles to biologic systems with the primary goal of optimizing clinical practice. We describe the efforts to use translational systems biology to develop an integrated framework to gain insight into the problem of acute inflammation. Progress in understanding inflammation using translational systems biology tools highlights the promise of this multidisciplinary field. Future advances in understanding complex medical problems are highly dependent on methodological advances and integration of the computational systems biology community with biologists and clinicians.

## Introduction: Inflammation Is a Complex System

Inflammation is a finely tuned, dynamic process, and its dysregulation underlies many complex diseases (e.g., sepsis, infectious disease, trauma, asthma, allergy, autoimmune disorders, transplant rejection, cancer, neurodegenerative diseases, obesity, and atherosclerosis). However, inflammation is not inherently detrimental. Inflammatory processes are required for immune surveillance, optimal repair, and regeneration after injury [1]–[3]. Inflammation is itself also a complex process, and like other complex systems, it has defied reductionist, linear definition [4]–[7]. Unfortunately, because a global definition or understanding of inflammation has not emerged from enormous data generated in basic biology, this deep dataset has not translated into mechanistic understanding sufficient to predict system behavior, and little in the way of effective therapies has emerged.

The discrepancy between biologic data and therapy has hampered the search for adequate clinical approaches in the settings of various inflammation-related disorders. More specifically and recognizing this discrepancy, the NIH Roadmap recently underscored the need to apply systems biology methods to the study of inflammation [8]. These methods include computational, mathematical, and engineering approaches (in silico methods) to facilitate translation of biomedical research.

**Figure 1:**
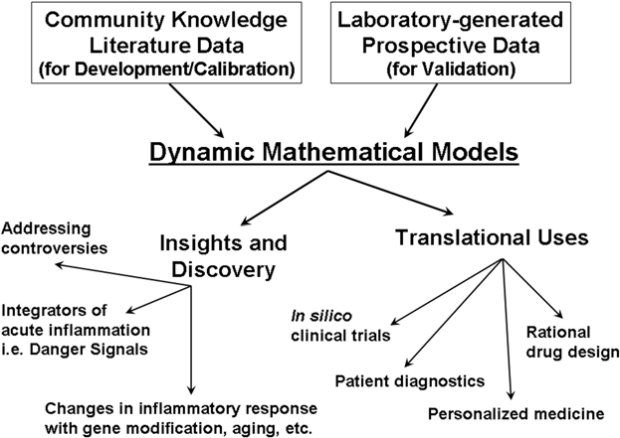
Overview of Translational Systems Biology. Pre-existing knowledge from the literature and newly generated information from wet lab experiments lead to the development of dynamic mathematical models. These computational simulations can then lead to both knowledge discovery, in the form of basic insights, and translational usage, such as in silico experiments and other engineering processes.

Systems biology has been defined in many ways [9]–[11], but generally is considered a global analytic approach to biologic data at the multiple scales of organization that characterize biologic systems, with the goal of identifying specific genetic and molecular signatures for improved diagnosis of disease [12]–[21]. Despite the richness of these approaches, there is still a relative paucity of techniques that transcend and describe the multiple scales and hierarchies of organization in a way that leads to effective therapeutic strategies. For instance, high throughput analyses (genomics, proteomics) have identified myriad factors and pathways involved in inflammation. However, the plethora of reductionist studies that these approaches were expected to replace have generated enormous amounts of data, but little in the way of translational insights necessary to use the data clinically. These high-throughput analyses rely on statistical methods for data interpretation and pattern analysis. We suggest that statistical methods must be augmented with dynamic modeling and simulation, along with mathematical tools from engineering, in order to address organization and behavior of dynamic complex disease processes. The goal of integrating these tools is to generate disease models that can be used for rapid translation, in areas as diverse as in silico clinical trials, diagnostics, and rational drug design. In addition, systems biology approaches can also yield fundamental insights into the mechanistic determinants of complex biologic processes. Further, we suggest the need to modify the way computational simulation is currently implemented to best address issues of direct clinical relevance, since to date computational and simulation technologies have been mostly utilized in the context of examining subcellular and cellular processes (Table 1, Figure 1) [9],[22],[23]. In order to translate the varied data streams from basic science into organism-level insights, the data must be organized into mechanistic, dynamical models to simulate higher-level (total organism) behavior and response to interventions. Translational systems biology requires that mathematical and modeling expertise be combined with expertise in various biological specialties and medical specialties.

**Table 1:** Comparison of Classical and Translational Systems Biology.

Classical Systems Biology	Translational Systems Biology
Basic insights are primary focus, i.e., “drilling down”	Translational insights are primary focus, i.e., “building up”
Models structured for greatest basic insights (cellular/molecular interactions, signal transduction pathways)	Models structured for clinical translational utility (in silico clinical trials, diagnostics, rational drug/device design)
Simulations designed for laboratory validation	Simulations designed for eventual clinical validation
“omics” studies applied to clinically relevant situations, and subsequently subjected to statistical analysis	Mechanistic simulations of whole-organism response guide “-omics” studies

Below, we detail the approaches used and salient findings observed thus far in the translational systems biology of inflammation. These studies have been applied to acute inflammatory responses including sepsis, trauma, hemorrhagic shock, and wound healing [7], [24]–[34], largely carried out under the aegis of the Society of Complexity in Acute Illness (SCAI, http://www.scai-med.org). Similar approaches are at the heart of many efforts, especially in industry, where there is an acute need for rational drug candidate discovery and improved efficiency in the transition from candidate compound to clinical trial [35]. However, the inflammation field is the first in which the translational systems biology framework has been a guiding principle applied in a systematic fashion. The primary methods of dynamic mathematical modeling utilized in these studies are agent-based modeling (ABM) [24],[25],[36] and equation-based modeling (EBM), the latter encompassing primarily ordinary differential equations (ODE) and partial differential equations (PDE) [26]–[31], [37]–[40]. The two forms of dynamic mathematical modeling have their respective strengths and weaknesses [7], but the utilization of both methods in the work described below demonstrates a pragmatic, goal-directed approach not tied to a particular modeling platform [7].

## Applications of Translational Systems Biology in Acute Inflammation: From Man to Mouse to Man

Translational systems biology has developed largely in response to the clinical challenge of sepsis. Sepsis is a syndrome resulting from massive, acute activation of the inflammatory response, traditionally in the setting of severe infection. Sepsis syndromes can often complicate trauma and/or hemorrhagic shock. In its most severe form, sepsis results in low blood pressure with insufficient perfusion of organs, and leads to multiple organ failure and death. Initial modeling studies were therefore focused on the pathophysiology of the acute inflammatory response to stress, and these studies pointed to common underlying processes generated in response to infection, injury, and shock. Later, as the modeling efforts matured to include the recovery phase of injury, the major insight gained was the link between the initial inflammatory response and subsequent recovery (healing). Here we present a history of how this field evolved around the seemingly intractable problem of sepsis, focusing on the rationale for the studies and insights derived from them.

Sepsis was the motivating clinical problem that led to mathematical modeling of inflammation. Intensive care physicians recognize that sepsis therapy has not changed substantially for decades despite an enormous amount of data generated from in vitro and animal studies, as well as from clinical studies [41]–[44]. The first approaches were designed to answer the question, “What are the dynamics of sepsis, and does our lack of therapies imply some yet undiscovered mediator of the syndrome?” In mainstream biology and biotechnology, this question motivates the ongoing search for a “magic bullet” to treat sepsis. The translational systems biology formulation of the question, though, was reworded to reflect a different philosophical approach to research, i.e., “Is the current state of knowledge insufficient to explain observed clinical behaviors?” Thus, the missing knowledge was assumed not to be a missing molecule or pathway, rather the missing knowledge was assumed to be an understanding of how all the various components involved in the sepsis response are organized and how they interact to generate a behavior. This question led to the use of ABM as a knowledge representation tool, constructed by reviewing the literature describing the molecular and cellular components of the acute inflammatory response. This model conceptualized inflammation as the interaction between endothelium (the single layer of cells that line blood vessels and delineate the vessel's lumen) and blood-borne inflammatory cells [24],[36], treating the whole organism as a gigantic endothelial cell surface over which inflammatory cells moved and interacted. Despite its abstraction, this model was able to qualitatively reproduce patterns of diverse clinical outcomes in sepsis [24]. In so doing, this model addressed certain controversial questions with respect to the dynamics of sepsis. The first of these was whether the anti-inflammatory response represented a subsequent and compensatory response to the initial pro-inflammatory response, or whether the pro- and anti-inflammatory responses were initiated concurrently. In the construction of ABM, no mechanistic evidence could be found to justify a defined “lag” in the anti-inflammatory response; published evidence at the time suggested that both pro- and anti-inflammatory cellular responses were triggered by the same stimuli. Instantiating those rules dynamically demonstrated that the anti-inflammatory response [represented by interleukin-10 (IL-10) levels] was indeed concurrent with the acute pro-inflammatory response [represented by interleukin-1 (IL-1) and tumor necrosis factor-α (TNF-α) levels]. The second interesting behavior uncovered by the model (now generally recognized) was that patients who suffered from the immune-suppressed phenotype of late-stage multiple organ failure and were susceptible to usually trivial nosocomial infections demonstrated sustained elevated markers of tissue damage and inflammation through two weeks of simulated time [29]–[30]. While this pattern seems obvious now, anti-cytokine drug trials had treatment protocols spanning only a single dose or a single day. Thus, this phenomenon (even if recognized) was not incorporated into the trial design, perhaps contributing to the failure of these candidate therapies.

The other initial modeling approach to sepsis took concepts used in ABM (restoring connections, representing concurrent processes and feedback loops) into qualitative EBM that described major phenomena in sepsis such as the role of anti-inflammatory responses and the paradoxical effects of pre-conditioning (in which two nominally pro-inflammatory stimuli can result in synergy or suppression relative to each stimulus alone) [37]–[39]. This work led to the creation of larger EBM calibrated with data obtained in the animal laboratory [27], with the premise that mathematical models require experimental validation and feedback between the models and experiments. The mouse studies were carried out for the express purpose of calibrating global EBM of acute inflammation, and confirmed the nearly simultaneous elaboration of the cytokines TNF-α and IL-10 after endotoxin (bacterial product) or trauma/hemorrhage. The base EBM was adjusted to match the initial conditions for both endotoxin challenge and trauma/hemorrhage. In each case, the subsequently generated, simulated cytokine profiles matched the wet lab patterns for each experimental preparation [27]. The model was sufficient to extrapolate in silico simulated data to prospective prediction of the threshold dose at which endotoxin would be lethal in mice, showing that information could be gleaned from the model beyond its calibration dataset [27].

Furthermore, in the process of calibrating this model to trauma/hemorrhage, we observed that the simulation of shock and global tissue ischemia/reperfusion was not reproducing the intensity or lethality of the inflammatory response in mice. The reason for the insufficiency of the model was that it did not reflect the practical issues in the animal model: it is impossible to have hemorrhage in the animal without some tissue injury. When a relatively trivial trauma (for extracting blood) was introduced into the simulation, the inflammatory mediator and lethality profiles seen in the mouse experiments were reproduced [30]. These findings along with parallel high throughput analysis of gene expression patterns (below) were incorporated into the in silico models, and ultimately led to the important conclusion that hemorrhage was not the driving response in acute inflammation after trauma/hemorrhage. Rather, the surgical trauma was the major signal initiating inflammation, leading to organ damage. These results show that the model development process alone can lead to interesting mechanistic insights into systems behavior. At the time, most specialists in the field thought that global ischemia/reperfusion was the predominant factor driving the inflammatory response to hemorrhagic shock.

We developed increasingly complex models of inflammation for quantitative prediction of circulating cytokine levels in rats, swine, and humans [28]. Other investigators, recognizing the clinical importance of these models, used EBM to describe the dynamics of bacterial growth in experimental pneumonia [31], and simulate the inflammatory response of influenza [34]. Different EBM was used to examine the nonlinear interactions between antibiotics and vaccination in the setting of anthrax infection [32]. EBM has also been used to study various facets of the inflammatory response to burn trauma, including the effects of resuscitation and cell-based therapy [45]–[52].

## The Golden Fleece: In Silico Clinical Trials for Sepsis

An important application of translational systems biology is use of simulations similar to those described above in the design and structuring of clinical trials. If successful, these tools have the potential to fundamentally transform the way clinical trials are conducted for acute inflammatory states such as sepsis and trauma. Simulations of clinical trial outcomes yield analysis of the patient subpopulations helped, harmed, or unaffected by a clinical intervention [26]. This type of sub-stratification of patients who deserve certain therapies is just now recognized as an important factor in clinical care. By incorporating genomic data, the simulations can be used to prospectively classify patients as appropriate recipients of a particular intervention (“personalized medicine”).

Translational systems biology has prompted development of methods to link extensive data on gene regulation and control to clinically relevant conditions. For example, EBM has been used to distinguish between the scale of gene expression (how much of each gene is expressed) and the scope of gene expression (number of pathways recruited) based on the severity of the insult [30]. The next step in modeling behavior of the inflammatory response, then, is likely to be incorporation of whole-genome expression levels coupled to network/pathway analysis in the model [13],[53], with in vivo validation in appropriate mouse models (such as mice with specific genes deleted [53]). In turn, the modified mathematical models that help define the role of a given gene product [29] could be further validated with in silico clinical trials [25],[26],[32] to determine whether the gene product is a valid therapeutic target.

Modeling has been integrated into actual trial design by utilizing the iterative process common to engineering projects, consisting of a knowledge/development loop between the real-world data and the simulation as the information from one source feeds into the next [28]. Models thus refined have already been used to assist in the analysis of ongoing clinical trials. In one case, the models produced a “virtual” placebo arm for an open-label Phase IV drug trial, providing an additional comparison for the actual Phase IV results and the preceding Phase III trial [54]. These examples used models to focus on efficacy of a single therapeutic intervention, and therefore were limited in scope, but the predictive value of the models is nonetheless evidence that translational systems biology approaches can have significant impact on the design and implementation of actual clinical therapies.

## After the Injury: The Link between Inflammation and Healing

We next turned our attention to the recovery phase, healing after acute inflammation. Wound healing (obviously part of trauma, but also often associated with sepsis) involves interaction of inflammatory mediators with mediators of the tissue remodeling and regenerative responses [1], [2], [55]–[60]. The early models of wound healing were built around epithelial proliferation and migration [61]–[70], followed by construction of a series of ABM incorporating interactions between regulators of inflammation and remodeling. These models were used to study necrotizing enterocolitis (NEC), a severe inflammatory intestinal disease of newborns [71],[72]. The regenerative process necessary both for reversal of the inflammatory response and for recovery from NEC involves migration of healthy enterocytes to sites of mucosal disruption [71]. PDE models of this process incorporated spatial effects such as diffusion of inflammatory agents, chemotaxis of both bacteria and inflammatory cells, and enterocyte migration [72]. The main insight gained by incorporation of these features into the model was that inflammation induced by an initial hypoxic/traumatic insult could be propagated by bacterial efflux through damaged epithelium.

To this point we have discussed models of acute inflammation, but chronic inflammation is also a major factor in impaired would healing, as in diabetes [2],[59],[73]. We modeled the relationship between inflammation and healing using ABM of diabetic foot ulcers [33]. Skin inflammation and healing in these wound models was calibrated to literature parameters. Single hypothesized, diabetes-related derangements in inflammatory mediators or factors involved in wound healing (namely, elevation of TNF-α and/or reduction of bioactive transforming growth factor-β1) were used in the model to predict delayed healing. The model recapitulated the beneficial effects of well-known therapies for diabetic foot ulcers—debridement and platelet-derived growth factor—and suggested novel therapies [33]. This particular example of computational simulation of a disease process highlights the potential to investigate results of both routine non-pharmacologic and pharmacologic interventions.

## Conclusions and Future Prospects

The literature on inflammation has yielded an enormous parts list of mediators and important information on how these mediators are linked to each other, but the entire range of inflammatory conditions has not been organized in a way that allows interpretation of data for design of clinical studies, or virtual manipulation of the system. This gap between experimentally derived details and application of knowledge to the bedside is being addressed by the translational systems biology community. This new field is developing binding methods, such as a syntactical modeling grammar that expresses hypotheses in formal logical syntax [74] to facilitate model construction and to improve the predictive clinical accuracy of models of inflammation.

Successful translation of models into practice requires further studies in several areas. For example, models are currently built and modified through a painstaking and time-consuming manual curation of the scientific literature. Certainly translational systems biology will benefit from automation of this process for mining data in a form that supports continuous updating of models. Similarly, non-mathematically trained clinicians often struggle with converting their intuitive biologic models into mathematic models using software developed for mathematicians. Translational systems biology needs improved software for facilitating translation of clinical knowledge into mathematical models.

If successful, these and other efforts will lead to model-driven design and testing of new therapies, clinical trials that are preceded by dry runs in silico, mathematical models to support diagnosis and therapy of critically ill patients, and outpatient plans developed using model-driven decisions along a continuum of care. Within a systems framework, these fragmented procedures can be treated as an integrated whole, in which biology-motivated mathematical models are used at every stage. The therapeutic utility of such approaches is treated with some skepticism now [75], but progress in understanding the complexity of inflammation should afford some optimism that, with input from the computational/systems biology community, translational systems biology efforts can lead to fundamental insights into optimal therapies.

## References

[1] Hart J (2002). Inflammation. 1: Its role in the healing of acute wounds.. J Wound Care.

[2] Hart J (2002). Inflammation. 2: Its role in the healing of chronic wounds.. J Wound Care.

[3] Bethea JR (2000). Spinal cord injury-induced inflammation: A dual-edged sword.. Prog Brain Res.

[4] Buchman TG, Cobb JP, Lapedes AS, Kepler TB (2001). Complex systems analysis: A tool for shock research.. Shock.

[5] Tjardes T, Neugebauer E (2002). Sepsis research in the next millennium: Concentrate on the software rather than the hardware.. Shock.

[6] Buchman TG (2002). The community of the self.. Nature.

[7] Vodovotz Y, Clermont G, Chow C, An G (2004). Mathematical models of the acute inflammatory response.. Curr Opin Crit Care.

[8] (2007). http://nihroadmap.nih.gov/inflammation/systemsbiology.asp.

[9] Kitano H (2002). Systems biology: A brief overview.. Science.

[10] Snoep JL, Westerhoff HV, Alberghina L, Westerhoff HV (2005). From isolation to integration, a systems biology approach for building the silicon cell.. Systems biology: Definitions and perspectives.

[11] Sauer U, Heinemann M, Zamboni N (2007). Genetics. Getting closer to the whole picture.. Science.

[12] Cobb JP, O'Keefe GE (2004). Injury research in the genomic era.. Lancet.

[13] Calvano SE, Xiao W, Richards DR, Felciano RM, Baker HV (2005). A network-based analysis of systemic inflammation in humans.. Nature.

[14] Cobb JP, Mindrinos MN, Miller-Graziano C, Calvano SE, Baker HV (2005). Application of genome-wide expression analysis to human health and disease.. Proc Natl Acad Sci U S A.

[15] Brownstein BH, Logvinenko T, Lederer JA, Cobb JP, Hubbard WJ (2006). Commonality and differences in leukocyte gene expression patterns among three models of inflammation and injury.. Physiol Genomics.

[16] Liu T, Qian WJ, Gritsenko MA, Xiao W, Moldawer LL (2006). High dynamic range characterization of the trauma patient plasma proteome.. Mol Cell Proteomics.

[17] Omenn GS (2006). Strategies for plasma proteomic profiling of cancers.. Proteomics.

[18] Ahrens CH, Wagner U, Rehrauer HK, Turker C, Schlapbach R (2007). Current challenges and approaches for the synergistic use of systems biology data in the scientific community.. EXS.

[19] Steinfath M, Repsilber D, Scholz M, Walther D, Selbig J (2007). Integrated data analysis for genome-wide research.. EXS.

[20] Tanke HJ (2007). Genomics and proteomics: The potential role of oral diagnostics.. Ann N Y Acad Sci.

[21] Kourtidis A, Eifert C, Conklin DS (2007). RNAi applications in target validation..

[22] Csete ME, Doyle JC (2002). Reverse engineering of biological complexity.. Science.

[23] Mo ML, Jamshidi N, Palsson BO (2007). A genome-scale, constraint-based approach to systems biology of human metabolism.. Mol Biosyst.

[24] An G (2001). Agent-based computer simulation and SIRS: Building a bridge between basic science and clinical trials.. Shock.

[25] An G (2004). In-silico experiments of existing and hypothetical cytokine-directed clinical trials using agent based modeling.. Crit Care Med.

[26] Clermont G, Bartels J, Kumar R, Constantine G, Vodovotz Y (2004). In silico design of clinical trials: A method coming of age.. Crit Care Med.

[27] Chow CC, Clermont G, Kumar R, Lagoa C, Tawadrous Z (2005). The acute inflammatory response in diverse shock states.. Shock.

[28] Vodovotz Y, Chow CC, Bartels J, Lagoa C, Prince J (2006). In silico models of acute inflammation in animals.. Shock.

[29] Prince JM, Levy RM, Bartels J, Baratt A, Kane JM (2006). In silico and in vivo approach to elucidate the inflammatory complexity of CD14-deficient mice.. Mol Med.

[30] Lagoa CE, Bartels J, Baratt A, Tseng G, Clermont G (2006). The role of initial trauma in the host's response to injury and hemorrhage: Insights from a comparison of mathematical simulations and hepatic transcriptomic analysis.. Shock.

[31] Ben David I, Price SE, Bortz DM, Greineder CF, Cohen SE (2005). Dynamics of intrapulmonary bacterial growth in a murine model of repeated microaspiration.. Am J Respir Cell Mol Biol.

[32] Kumar R, Chow CC, Bartels J, Clermont G, Vodovotz Y (2007). A mathematical simulation of the inflammatory response to anthrax infection.. Shock.

[33] Mi Q, Rivière B, Clermont G, Steed DL, Vodovotz Y (2007). Agent-based model of inflammation and wound healing: Insights into diabetic foot ulcer pathology and the role of transforming growth factor-b1.. Wound Rep Reg.

[34] Hancioglu B, Swigon D, Clermont G (2007). A dynamical model of human immune response to influenza A virus infection.. J Theor Biol.

[35] Food and Drug Administration (2004). Innovation or stagnation: Challenge and opportunity on the critical path to new medical products..

[36] An G, Lee I (2000). Complexity, emergence and pathophysiology: Using agent based computer simulation to characterize the non-adaptive inflammatory response (Manuscript # [344]).. http://www.interjournal.org.

[37] Kumar R, Clermont G, Vodovotz Y, Chow CC (2004). The dynamics of acute inflammation.. J Theoretical Biol.

[38] Reynolds A, Rubin J, Clermont G, Day J, Vodovotz Y (2006). A reduced mathematical model of the acute inflammatory response: I. Derivation of model and analysis of anti-inflammation.. J Theor Biol.

[39] Day J, Rubin J, Vodovotz Y, Chow CC, Reynolds A (2006). A reduced mathematical model of the acute inflammatory response: II. Capturing scenarios of repeated endotoxin administration.. J Theor Biol.

[40] Upperman JS, Lugo B, Camerini V, Yotov I, Rubin J (2007). Mathematical modeling in NEC—A new look at an ongoing problem.. J Pediatr Surg.

[41] Freeman BD, Natanson C (2000). Anti-inflammatory therapies in sepsis and septic shock.. Expert Opin Investig Drugs.

[42] Marshall JC (2000). Clinical trials of mediator-directed therapy in sepsis: What have we learned?. Intensive Care Med.

[43] Cunneen J, Cartwright M (2004). The puzzle of sepsis: Fitting the pieces of the inflammatory response with treatment.. AACN Clin Issues.

[44] Esmon CT (2004). Why do animal models (sometimes) fail to mimic human sepsis?. Crit Care Med.

[45] Roa LM, Gomez-Cia T, Cantero A (1988). Analysis of burn injury by digital simulation.. Burns Incl Therm Inj.

[46] Roa L, Gomez-Cia T, Cantero A (1990). Pulmonary capillary dynamics and fluid distribution after burn and inhalation injury.. Burns.

[47] Bert J, Gyenge C, Bowen B, Reed R, Lund T (1997). Fluid resuscitation following a burn injury: Implications of a mathematical model of microvascular exchange.. Burns.

[48] Rosinski M, Yarmush ML, Berthiaume F (2004). Quantitative dynamics of in vivo bone marrow neutrophil production and egress in response to injury and infection.. Ann Biomed Eng.

[49] Feng Q, Zhao-Yan H, Zheng-Kang Z, Li-Xing S (2005). The establishment of the mathematical model of the 2^nd^ degree burn injury of human tissues and its application.. Conf Proc IEEE Eng Med Biol Soc.

[50] Mercer GN, Sidhu HS (2005). Modeling thermal burns due to airbag deployment.. Burns.

[51] Lv YG, Liu J, Zhang J (2006). Theoretical evaluation of burns to the human respiratory tract due to inhalation of hot gas in the early stage of fires.. Burns.

[52] Denman PK, McElwain DL, Harkin DG, Upton Z (2007). Mathematical modelling of aerosolised skin grafts incorporating keratinocyte clonal subtypes.. Bull Math Biol.

[53] Gilchrist M, Thorsson V, Li B, Rust AG, Korb M (2006). Systems biology approaches identify ATF3 as a negative regulator of Toll-like receptor 4.. Nature.

[54] Chang S, Baratt A, Clermont G, Planquois J-M, Yan SB (2006). Mathematical model predicting outcomes of sepsis patients treated with Xigris(R): ENHANCE trial.. Shock.

[55] Goldring SR (2003). Inflammatory mediators as essential elements in bone remodeling.. Calcif Tissue Int.

[56] Guilak F, Fermor B, Keefe FJ, Kraus VB, Olson SA (2004). The role of biomechanics and inflammation in cartilage injury and repair.. Clin Orthop.

[57] Ramadori G, Saile B (2004). Inflammation, damage repair, immune cells, and liver fibrosis: Specific or nonspecific, this is the question.. Gastroenterology.

[58] Redd MJ, Cooper L, Wood W, Stramer B, Martin P (2004). Wound healing and inflammation: Embryos reveal the way to perfect repair.. Philos Trans R Soc Lond B Biol Sci.

[59] Diegelmann RF, Evans MC (2004). Wound healing: An overview of acute, fibrotic and delayed healing.. Front Biosci.

[60] Thornton FJ, Schaffer MR, Barbul A (1997). Wound healing in sepsis and trauma.. Shock.

[61] Murray JD (1989). Mathematical biology.

[62] Sherratt JA, Murray JD (1990). Models of epidermal wound healing.. Proc Biol Sci.

[63] Tranquillo RT, Murray JD (1992). Continuum model of fibroblast-driven wound contraction: Inflammation-mediation.. J Theor Biol.

[64] Tranquillo RT, Murray JD (1993). Mechanistic model of wound contraction.. J Surg Res.

[65] Cook J (1995). A mathematical model for dermal wound healing: Wound contraction and scar formation [dissertation].

[66] Olsen L, Sherratt JA, Maini PK (1995). A mechanochemical model for adult dermal wound contraction and the permanence of the contracted tissue displacement profile.. J Theor Biol.

[67] Dallon JC, Sherratt JA, Maini PK (2001). Modeling the effects of transforming growth factor-beta on extracellular matrix alignment in dermal wound repair.. Wound Repair Regen.

[68] Sherratt JA, Dallon JC (2002). Theoretical models of wound healing: Past successes and future challenges.. C R Biol.

[69] Walker DC, Hill G, Wood SM, Smallwood RH, Southgate J (2004). Agent-based computational modeling of epithelial cell monolayers: Predicting the effect of exogenous calcium concentration on the rate of wound closure.. IEEE Trans Nanobioscience.

[70] Walker DC, Southgate J, Hill G, Holcombe M, Hose DR (2004). The epitheliome: Agent-based modelling of the social behaviour of cells.. Biosystems.

[71] Hackam DJ, Upperman JS, Grishin A, Ford HR (2005). Disordered enterocyte signaling and intestinal barrier dysfunction in the pathogenesis of necrotizing enterocolitis.. Semin Pediatr Surg.

[72] Upperman JS, Lugo B, Camerini V, Yotov I, Rubin J (2006). Mathematical modeling in NEC—A new look at an ongoing problem.. J Pediatr Surg.

[73] Sweitzer SM, Fann SA, Borg TK, Baynes JW, Yost MJ (2006). What is the future of diabetic wound care?. Diabetes Educ.

[74] An G (2006). Concepts for developing a collaborative in silico model of the acute inflammatory response using agent-based modeling.. J Crit Care.

[75] Marshall JC (2004). Through a glass darkly: The brave new world of in silico modeling.. Crit Care Med.

